# TWIST1 Heterodimerization with E12 Requires Coordinated Protein Phosphorylation to Regulate Periostin Expression

**DOI:** 10.3390/cancers11091392

**Published:** 2019-09-18

**Authors:** Svetlana A. Mikheeva, Nathan D. Camp, Lei Huang, Antrix Jain, Sung Yun Jung, Naze G. Avci, Mari Tokita, Alejandro Wolf-Yadlin, Jing Zhang, Stephen J. Tapscott, Robert C. Rostomily, Andrei M. Mikheev

**Affiliations:** 1 Department of Neurosurgery, Houston Methodist Research Institute, Houston, TX 77030, USA; 2 Department of Neurological Surgery, University of Washington, Seattle, WA 98195, USA; 3 Institute for Stem Cell and Regenerative Medicine, University of Washington, Seattle, WA 98109, USA; 4 Department of Genome Sciences, University of Washington, Seattle, WA 98195, USA; 5 Seattle Children’s Research Institute, Seattle, WA 98101, USA; 6 Department of Systems Medicine & Bioengineering, Houston Methodist Research Institute, Houston, TX 77030, USA; 7 Baylor College of Medicine, Houston, TX 77030, USA; 8 Division of Medical Genetics, University of Washington, Seattle, WA 98195, USA; 9 Seattle Genetics, Bothell, WA 98021, USA; 10 Division of Neuropathology, Department of Pathology, University of Washington, Seattle, WA 98195, USA; 11 Fred Hutchinson Cancer Research Center, Seattle, WA 98109, USA

**Keywords:** TWIST1, dimerization, invasion, glioblastoma, periostin

## Abstract

Diffuse invasion into adjacent brain matter by glioblastoma (GBM) is largely responsible for their dismal prognosis. Previously, we showed that the TWIST1 (TW) bHLH transcription factor and its regulated gene periostin (*POSTN*) promote invasive phenotypes of GBM cells. Since TW functional effects are regulated by phosphorylation and dimerization, we investigated how phosphorylation of serine 68 in TW regulates TW dimerization, *POSTN* expression, and invasion in glioma cells. Compared with wild-type TW, the hypophosphorylation mutant, TW(S68A), impaired TW heterodimerization with the E12 bHLH transcription factor and cell invasion in vitro but had no effect on TW homodimerization. Overexpression of TW:E12 forced dimerization constructs (FDCs) increased glioma cell invasion and upregulated pro-invasive proteins, including POSTN, in concert with cytoskeletal reorganization. By contrast, TW:TW homodimer FDCs inhibited POSTN expression and cell invasion in vitro. Further, phosphorylation of analogous PXSP phosphorylation sites in TW:E12 FDCs (TW S68 and E12 S139) coordinately regulated *POSTN* and *PDGFRa* mRNA expression. These results suggested that TW regulates pro-invasive phenotypes in part through coordinated phosphorylation events in TW and E12 that promote heterodimer formation and regulate downstream targets. This new mechanistic understanding provides potential therapeutic strategies to inhibit TW-POSTN signaling in GBM and other cancers.

## 1. Introduction

Glioblastoma (GBM), the most common and malignant adult primary brain cancer, is essentially incurable with median survivals of only 12–16 months from diagnosis. Little progress has been realized in outcomes over four decades underscoring the critical need for new therapeutic approaches. One potential therapeutic target is mechanisms, which promote mesenchymal transition [1,2]. Mesenchymal changes in GBM, which are similar to epithelial mesenchymal transition (EMT) in other cancers, promote canonical features of malignancy including cell invasion, cancer cell stemness, and treatment resistance contributing to poor prognoses [3,4,5,6]. Among the mechanisms that drive EMT, TWIST1 (TW), a bHLH transcription factor and master regulator of EMT, and its transcriptional target periostin (POSTN), a secreted matrix protein, play prominent roles in cancer [7,8]. In GBM we demonstrated that TW promotes mesenchymal change by regulating invasion, GBM stem cell self-renewal and tumorigenicity [9,10]. Expression of POSTN is regulated by, and directly correlates with, TW expression in human gliomas, and like TW, promotes invasion and tumorigenicity of GBM cells [11]. While these observations support the potential therapeutic relevance of TW-POSTN signaling, the mechanisms by which TW may regulate POSTN expression in GBM and other cancers are not known. Potential candidate mechanisms include active site-specific TW phosphorylation and TW dimerization motifs with functional relevance in development and cancer.

Site-specific TW phosphorylation fundamentally alters developmental phenotypes and most commonly confers increased malignant phenotypes related to invasion and EMT [12,13]. For example, the phosphorylation of serine 68 in TW profoundly impacts the malignant EMT related phenotypes in breast carcinoma. Using an S68A TW hypo-phosphomutant Hong et al. showed that maximal TW mediated invasion of breast carcinoma cells and activation of EMT phenotypes was dependent on phosphorylation of TW S68 [14]. Similarly, Sun et al. showed that TW mediated activation of EMT and invasion of breast carcinoma cells was negatively regulated through SCP 1 (small C-terminal domain phosphatase) dependent dephosphorylation of TW S68 [15]. In addition to site-specific phosphorylation, TW function is mediated by its choice of dimerization partners required for DNA binding and transcriptional regulation. Formed by HLH domain interactions, TW dimerizes with itself to form homodimers (TW:TW) or other bHLHs, such as E12 [13,16], to generate heterodimers (TW:E12). Different TW dimer motifs have profound functional impacts on normal mesodermal developmental processes including myogenesis, cardiac development, and cranial suture formation [17,18,19,20]. In breast and prostate cancer models, TW:E12 promotes increased malignancy [21,22], whereas TW homodimerization is associated with increased malignancy in pancreatic carcinoma [23]. Thus, site-specific TW phosphorylation and TW dimerization motifs can function as robust phenotypic switches regulating development and cancer malignancy.

Multiple lines of evidence suggest potential interactions between site-specific TW phosphorylation and TW dimerization motifs in regulating *POSTN* expression and mesenchymal phenotypes. For instance, developmental models demonstrate robust phenotypes dependent on TW phosphorylation mediated regulation of TW dimerization motifs [13,20]. Of importance, POSTN expression and functional phenotypes in the osteogenic front of developing cranial sutures are differentially regulated by specific TW dimer motifs [17]. Together these observations support functionally relevant mechanistic links between TW phosphorylation, dimerization and regulation of *POSTN* expression. However, similar mechanisms have not been established in cancer studies. Hong et al. found no evidence for an association between TW S68 phosphorylation and TW:E12 heteromdimerization in a yeast two-hybrid assay [14]. In a prostate carcinoma model malignant phenotypes generated by phospho-mimetic TW were highly correlated with those of a TW:E12 tethered dimer but no direct connection between phosphorylation and dimer formation was shown [21]. In pancreatic cancer TW phosphorylations at S123, T148 and S184 were associated with preferential TW homodimerization and EMT phenotypes, but functional activity of the TW homodimer was not directly studied [23]. Collectively these observations support the importance of TW phosphorylation dependent TW dimerization but direct validation and functional comparisons of TW dimers with regard to invasion and regulation of *POSTN* expression are lacking.

Therefore, here we sought to test the hypothesis that TW mediates mesenchymal changes and *POSTN* expression through site-specific TW phosphorylation dependent regulation of TW dimerization motifs. To test this hypothesis we studied the role of TW S68 phosphorylation in regulating TW dimerization motifs and POSTN expression using hypo-phosphorylation TW mutants and forced TW:TW homodimer or TW:E12 heterodimer constructs in GBM cells. Our results demonstrated a novel mechanism whereby coordinated TW and E12 phosphorylation are required for preferential formation of pro-invasive TW:E12 heterodimers that drive maximal transcriptional activation of *POSTN* expression. This new understanding may provide new targets for intervention that could be leveraged to inhibit the TW-POSTN signaling axis in GBM and other cancers.

## 2. Results

### 2.1. TW S68 Phosphorylation Detected in Human GBM and GBM Cells Promotes Invasion

**Figure 1:**
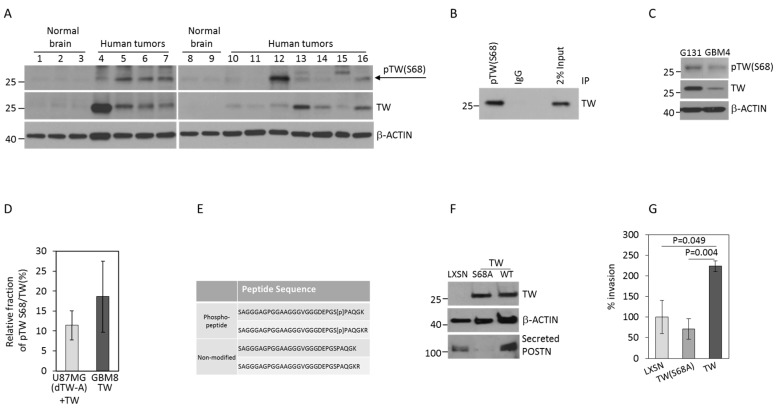
Identification and functional characterization of phosphorylation at S68 residue in TW. (**A**) Detection of TW S68 phosphorylation and total TW in patient GBM samples compared to normal brains. (**B**) TW S68 phosphorylation at the endogenous level in T98G cells detected by immunoprecipitation with pTW S68 antibody followed by detection using a total TW antibody. As a negative control non-specific same isotype IgG was used. Inputs were 2% of total proteins used for immunoprecipitation. (**C**) Expression of pTW S68 and total TW in primary glioma stem cells GBM4 and G131. (**D**) Relative fraction of pTW S68 in total amount of TW immunoprecipitated from U87MG (dTW-A) and GBM8 cells with TW overexpression. The percent of pTWS68 is defined as area under the curve of phospho-peptide divided by sum of pTWS68 + Non-phospho-peptide and averaged from three biological replicates. (**E**) Non-modified and phosphopeptides detected in U87MG (dTW-A) and GBM8 cells with TW overexpression and used for calculation of S68 phosphorylation fraction in panel D (see Figure S2). (**F**) Confirmation of comparable levels of TW and TW(S68A) mutant overexpression in T98G cells and corresponding alteration of POSTN secretion. (**G**) Increased invasion of T98G TW cells in a matrigel invasion assay is completely reversed in T98G cells expressing the TW(S68A) mutant.

To establish the potential relevance of TW S68 phosphorylation for GBM functional phenotypes we first confirmed its presence in patient-derived GBM samples using a S68 phospho-specific TW antibody and normal brain samples (Figure 1A). Higher levels of pTWS68 and total TW are detected in tumors compared to normal brain. However, levels of pTWS68 in tumors do not always correlate with expression levels of the total TW. We then confirmed the presence of TW S68 phosphorylation at the endogenous level in glioma cells and patient-derived GBM cell lines to establish its potential relevance for GBM tumor cell specific phenotypes. We performed immunoprecipitation using phospho-TW S68 antibody and detected phosphorylated protein form with total TW antibody in T98G cells (Figure 1B). This experiment demonstrated TW phosphorylation at the endogenous levels in glioma cells and verified the specificity of the pTWS68 antibody by detecting immunoprecipitated protein with an unrelated TW antibody. Subsequently we detected pTWS68 expression in glioma primary cells (GBM4 and G131) using Western blot (Figure 1C).

Next we used phosphoproteomics to estimate relative fraction of TW S68 phosphorylation in glioma cells. We used cells with exogenous TW overexpression in GBM8 glioma stem cells (undetectable endogenous TW) and U87MG cells (high endogenous TW) with CRISPR mediated TW knockout and subsequent overexpression of exogenous TW (U87-dTW-A + TW) [24]. The choice of TW deleted cells re-overexpressing TW was based on our previous validation of their increased tumorigenicity in vivo [24] and to determine whether this functional phenotype correlated with detection of pTWS68. Overexpression of TW provided a convenient platform to perform reliable relative quantification of pTWS68 phosphorylation. Representative mass spectra demonstrating detection of the S68 phosphosite are shown in Figure S1A,B for (U87dTW-A + TW) and GBM8 TW, respectively. TW S68 phosphorylation after overexpression was confirmed using Western blot (Figure S1C,D). The proportion of TW S68 phosphorylation in U87 (dTW-A + TW) and GBM8 TW cells was estimated to be approximately 12% and 18%, respectively (Figure 1D,E and Figure S2).

To establish the functional relevance of the S68 phosphosite for GBM cells we then over-expressed an S68 hypophosphomutant (TW(S68A)) or WT TW in T98G cells and demonstrated similar expression levels of both proteins by Western blot (Figure 1F). We previously demonstrated that TW overexpression activates POSTN expression in U87MG and GBM4 cells [24]. Consistent with previous results overexpression of WT TW resulted in activation of POSTN secretion, however, overexpression of the TW(S68A) mutant inhibited POSTN secretion. The absence of phosphorylation in TW(S68A) cells was confirmed using a pTW S68-specific antibody, as demonstrated in Figure S1C,D. We compared in vitro invasion of T98G empty vector control cells (T98G LXSN) with TW and TW(S68A) expressing cells. Invasion of T98G cells over-expressing the TW(S68A) hypo-phosphorylation mutant was markedly reduced compared with T98G cells over-expressing WT TW (Figure 1G) and correlated with POSTN secretion. We also generated and tested TW(S68E) phospho-mimicking mutant. However, this mutant failed to activate POSTN expression and cell invasion in vitro in T98G cell (not shown). Our results are consistent with previous observations [14] demonstrating that the TW(S68E) mutant does not mimic S68 phosphorylation in multiple assays. We, therefore, did not use the TW(S68E) mutant in subsequent experiments. Together these studies indicated that TW phosphorylation at S68 is a common post-translation modification in human gliomas and consistent with prior observations in breast carcinoma cells [14], the capacity for S68 phosphorylation and activation of POSTN may be critical for TW mediated glioma cell invasion.

### 2.2. Serine 68 Phosphorylation in TW Promotes Interaction with the E12 Binding Partner

**Figure 2:**
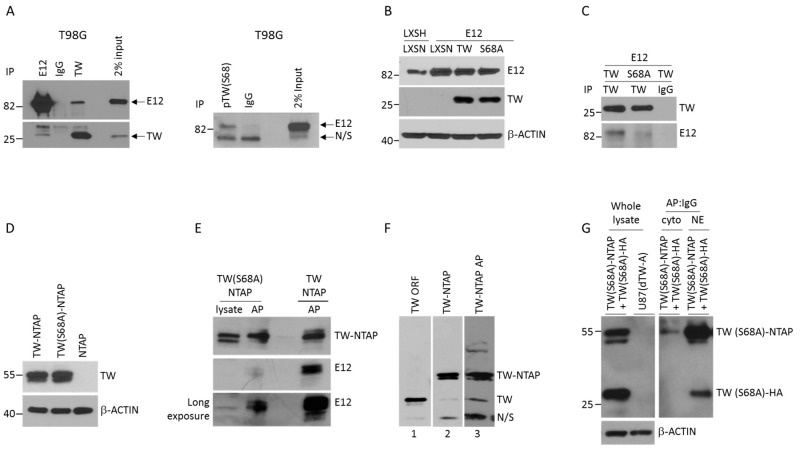
S68 phosphorylation promotes TW heterodimerization with E12 but is not required for TW homodimerization. (**A**) Co-immunoprecipitation (left) of endogenous E12 and TW. Antibodies (mouse origin) used for immunoprecipitation are shown on the top. Non-specific IgG is of the same IgG1 isotype. Inputs were 2% of total proteins used for immunoprecipitation. Western blot was probed with rabbit antibody to detect E12 and TW. Co-immunoprecipitation (right) of endogenous pTW S68 and E12. Immunoprecipitates of pTW S68 were detected with mouse E12 antibody. N/S—non-specific signal. (**B**) Comparable levels of E12, WT and S68A mutant TW expression confirmed by western blot of T98G cells stably transduced with E12 cloned in LXSH or empty LXSH followed by transduction with pLXSN vector, TW, or TW(S68A). (**C**) Differential detection of E12 after TW immunoprecipitation from nuclear extracts of T98G cells expressing WT or S68A TW. IgG- nonspecific antibody control. (**D**) Comparable levels of TW and TW(S68A) protein expressed in T98G cells transduced with an empty NTAP-LXSN vector, TW-, or TW(S68A)-NTAP cloned in pLXSN. (**E**) Enrichment of endogenous E12 in T98G cells expressing WT TW-NTAP versus TW(S68A)-NTAP after NTAP affinity purification (AP) of nuclear lysates. Nuclear lysate from cells transduced with TW (S68A)-NTAP was loaded to monitor protein expression before AP. The long exposure panel demonstrates endogenous levels of E12 proteins before AP. (**F**) Detection of endogenous TW as a homodimer binding partner with TW-NTAP in T98G cells. After AP of nuclear extracts from T98G TW-NTAP expressing cells (lane 3 versus 2), co-purified endogenous TW is detected at the same molecular weight (TW) as an open reading frame (ORF) wild-type TW over-expressed in T98G (lane 1). N/S- non-specific band. (**G**) TW homodimer formation does not require S68 phosphorylation. Cells were co-transduced with tagged TW(S68A)-NTAP and TW(S68A)-HA. Co-expression of both transgenes was confirmed using TW antibody. Lysates from TW null U87 (dTW-A) cells were used as a negative control (left panel). AP was performed on cytoplasmic and nuclear fractions followed by probing with HA antibody to detect the TW(S68A)-HA and secondary IgG to detect the TW-NTAP fusion protein harboring protein A (right panel).

TW functions as a homodimer or heterodimer, the latter most commonly generated through TW binding to E12. TW dimerization motifs profoundly influence TW regulated phenotypes with the TW:E12 heterodimer most often associated with increased malignancy [21,22]. To establish the role of TW:E12 heterodimers in GBM we first confirmed TW interaction with E12 in GBM cells. Using reciprocal co-immunoprecipitation (co-IP), endogenous E12 and TW interaction was detected in T98G cells in vitro (Figure 2A, left panel). We further found that endogenous phosphorylated TW interacts with E12 (Figure 2A, right panel). Since the role of specific phosphorylation sites in regulating TW:E12 dimerization motifs has not been established, we studied the effects of TW S68A hypo-phosphomutant expression on interactions with E12. In T98G cells we co-expressed E12 and TW WT or TW(S68A) and achieved comparable levels of E12, TW and TW(S68A) expression (Figure 2B). Using co-IP we demonstrated reduced detection of E12 interacting with TW(S68A) compared to wild-type TW (Figure 2C). To confirm this finding we generated TW-NTAP and TW(S68A)-NTAP constructs for affinity purification (AP). Equal amounts of tagged TW were detected in each cell type expressing TW-NTAP or TW(S68A)-NTAP (Figure 2D). After AP of the TW-NTAP proteins we observed a marked enrichment of endogenous E12 in nuclear fractions of T98G TW-NTAP compared with T98G TW(S68A)-NTAP cells (Figure 2E). Together these observations indicated that the detection of TW:E12 interactions was markedly reduced in the absence of TW S68 phosphorylation. To determine whether TW can also form homodimers in glioma cells we performed an affinity purification of nuclear extracts from T98G cells overexpressing TW-NTAP. Western blot analysis demonstrated the enrichment of endogenous wild-type TW after AP (Figure 2F) indicating that TW can form homodimers in T98G cells. Due to low yields we could not directly confirm the S68 phosphorylation status of the homodimeric TW binding partner. However, by co-expression of TW(S68A)-NTAP and HA tagged TW(S68A) and affinity purification of TW(S68A)-NTAP we showed that TW(S68A) phosphomutant is accumulated in the nuclei and forms TW homodimers (Figure 2G). Therefore, phosphorylation of S68 specifically promoted TW:E12 heterodimer formation and was not required for the formation of TW homodimers. Further studies are required to determine whether phosphorylation at S68 actively inhibits TW homodimerization or homodimerization is insensitive to S68 phosphorylation.

### 2.3. TW:E12 Heterodimers and TW:TW Homodimers Differentially Regulate Expression of Periostin (POSTN), Glioma Cell Adhesion, and Invasion 

Arguably, the most consistent functional effect of TW expression in all cancers including glioma is to promote invasion [5,9,10,25]. Pro-invasive phenotypes of TW over-expressing glioma cells are associated with increased POSTN expression, increased adhesion to fibronectin (FN), alterations in F-actin organization and increased phospho-FAK expression [10]. We observed that inhibition of GBM cell tumorigenicity after *POSTN* knockdown was associated with reduced FAK expression and membrane localization [11] implicating FAK in TW-POSTN signaling axis. *POSTN* in mouse cells is a direct transcriptional target of TW [26] that phenocopies the pro-invasive function of TW in glioma cells [10,11,24]. Therefore, we investigated whether TW heterodimerization with E12 and TW homodimers regulate *POSTN* expression.

**Figure 3:**
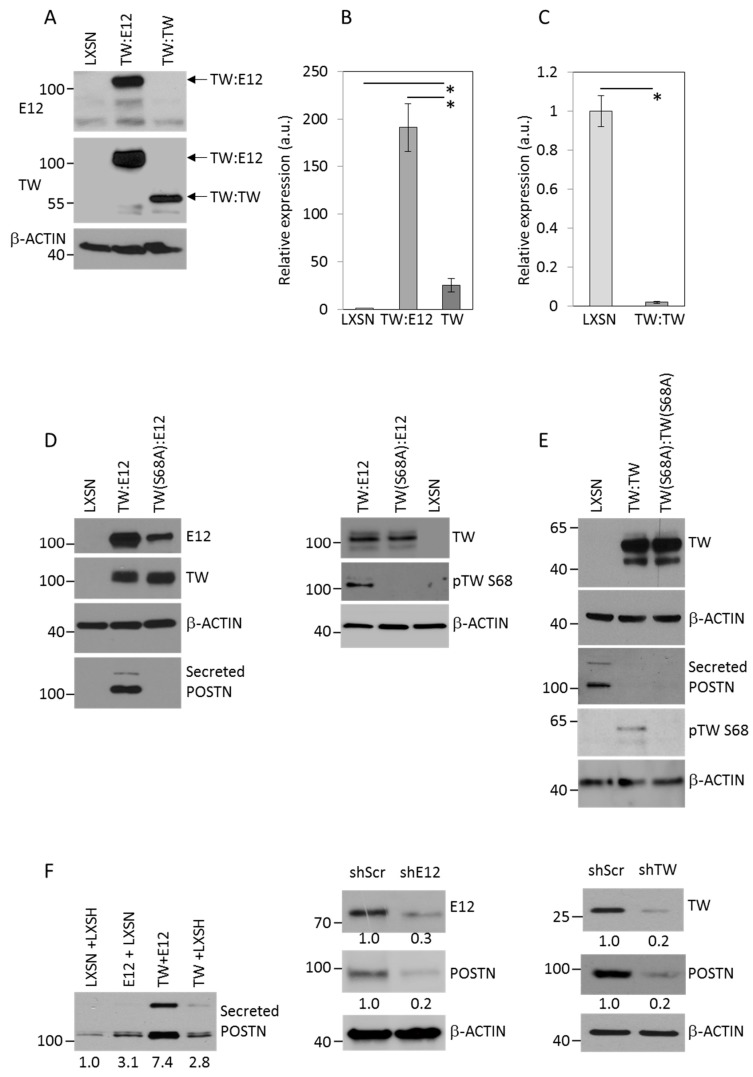
Effect of TW dimer motifs and S68 phosphorylation on POSTN expression. (**A**) Confirmation of FDC expression in T98G whole cell lysates. Parallel membranes were probed with E12 (top) or TW (middle) antibodies. (**B**) Marked increase of *POSTN* mRNA expression in T98G cells expressing TW:E12 heterodimer or TW monomer versus LXSN vector control (* *p* < 0.0001). (**C**) Inhibition of POSTN mRNA expression in TW:TW homodimer expressing cells compared to LXSN control (* *p* < 0.0001). mRNA levels were measured by qRT-PCR. (**D**) Effect of TW:E12 versus TW (S68A):E12 FDCs on secreted POSTN in T98G cells. FDC expression is confirmed using Tw and E12 antibodies. Secreted POSTN is detected after 24 h in serum free cell culture media and normalized by cell number. Independent experiment was performed to detect TW heterodimers and pTWS68 phosphorylation in WT and S68A mutant FDCs using total TW antibody or pTW S68 antibody, respectively (right panel). (**E**) Expression of TW homodimers is confirmed using TW antibody. Detection of secreted POSTN and pTW S68 in cells expressing TW:TW, TW(S68S):TW(S68A) or empty vector (LXSN) as described for panel D. (**F**) Alteration of TW and E12 expression or knockdown results in alteration of POSTN expression. Equal cell numbers of T98G cells overexpressing indicated vectors were plated at sub-confluency for 24 h, washed with PBS and switched to a serum free media. Conditioned media was collected after 24 hours for POSTN detection and normalized by cell number. Confirmations of TW and/or E12 overexpression are shown in Figure 2B,C. E12 or TW were knockdown using shRNA. Levels of endogenous E12, TW and POSTN were detected using Western blot. Relative levels of expression normalized by actin are shown under the panel. Expression levels in cells transduced with shScr are accepted as 1. Secreted POSTN migrates slightly slower compared to intracellular POSTN.

Therefore, to determine the functional significance of specific TW dimerization motifs we expressed forced dimerization constructs (FDCs) of TW:E12 and TW:TW in T98G cells. Expression of the FDCs was confirmed in T98G cells by Western blot (Figure 3A). Next we compared *POSTN* mRNA levels in T98G cells expressing TW or TW:E12 and TW:TW FDCs compared with empty vector controls. TW expression alone increased *POSTN* mRNA levels by ~25-fold while TW:E12 FDCs generated a ~190-fold induction of *POSTN* mRNA compared with the empty vector control (Figure 3B). By contrast, expression of TW:TW FDCs dramatically reduced *POSTN* mRNA expression to nearly undetectable levels compared to vector control cells (Figure 3C). We showed above that the maximal TW dimerization with E12 required the presence of a wild-type TW S68 phosphorylation (Figure 2C,E). To determine whether upregulation of POSTN by TW:E12 FDCs also required the presence of the WT TW S68 phosphorylation we generated FDCs comprised of the TW(S68A) mutant and E12 (TW(S68A):E12), and determined their effects on POSTN expression compared with TW:E12 FDCs. Consistent with mRNA data, secreted POSTN was readily detected in conditioned media from T98G TW:E12 FDC cells but remained at very low levels in conditioned media of TW(S68A):E12 cells (Figure 3D). Further, the detection of POSTN in TW:E12 cells correlated with the phosphorylation of TW S68. As expected, secreted POSTN could not be detected in conditioned media from T98G cells expressing TW:TW or TW(S68A):TW(S68A) FDCs although some TW S68 phosphorylation was detected in TW:TW FDCs (Figure 3E). Together, these observations suggested that the upregulation of POSTN by TW requires both TW:E12 dimer formation and TW S68 phosphorylation. To support the physiologic relevance of data acquired with TW:E12 FDCs we assayed the effects of altering the expression of freely interacting TW and E12 monomers on expression of POSTN. While POSTN detection in conditioned media was increased in T98G cells expressing TW or E12 alone, maximal expression of POSTN was achieved with co-expression of TW and E12 and knockdown of either TW or E12 alone resulted in significant reduction of POSTN (Figure 3F). These results indicated that stoichiometric relationships favoring TW and E12 interactions, and, presumably, TW:E12 dimerization, enhance POSTN expression similar to the effects imposed through forced TW and E12 interactions by FDCs.

**Figure 4:**
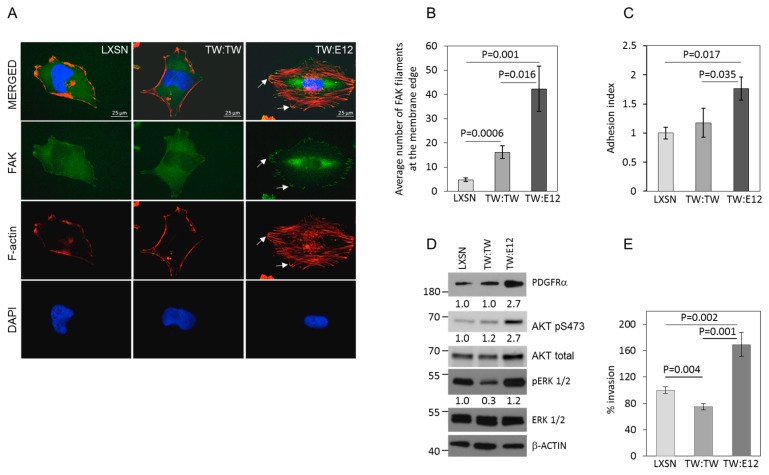
Differential regulation of cellular phenotypes by expression of TW:E12 and TW:TW forced dimer constructs (FDCs). (**A**) Representative confocal images of FAK immunocytochemistry and phalloidin staining to detect F-actin in T98G cells expressing LXSN control vector, TW:TW and TW:E12 FDCs. FAK localized at membrane (arrows) appears to be increased in TW:E12-expressing cells and co-localize with F-actin filaments. (**B**) Quantification of FAK at the membrane edge. *P* values are shown. (**C**) Cells expressing TW:E12 FDC show increased adhesion to Fibronectin 1 compared to TW:TW FDC or vector control T98G cells. Data shown as fold change over mean adhesion in control accepted as 1. *P*-values are shown. (**D**) Dimer dependent activation of pro-invasive proteins. Overexpression of TW:E12 heterodimer results in activation of PDGFRa protein expression and pAKT S473 phosphorylation. Overexpression of TW:TW homodimers results in inhibition of ERK1/2 phosphorylation. Relative expression normalized by actin is shown under the corresponding panel. Expression in cells transduced with empty vector accepted as 1. (**E**) Matrigel invasion assay showing TW:E12 heterodimer FDC expression markedly increases invasion while TW:TW homodimer FDC expression slightly decreases invasion compared with vector controls in T98G cells.

Further, consistent with our previously published results [10,11] in adherent cell cultures, TW:E12 FDC expression resulted in significant increase in membrane localization of FAK and F-actin stress fiber formation compared with cells expressing the control vector or TW:TW FDC (Figure 4A,B) In TW:E12 overexpressing cells membrane FAK foci co-localized with F-actin filaments. More modest increases in FAK membrane localization were detected in TW:TW cells compared with controls and lacked changes in F-actin stress fiber formation seen in TW:E12 cells. Consistent with alteration of FAK and F-actin expression we observed increased adhesion to fibronectin of cells with TW:E12 FDC expression (Figure 4C).

We next examined whether changes in TW regulated pro-invasive downstream targets and pathways including PDGFRa, AKT, and ERK1/2 reflected the dimer specific invasive phenotypes. Our results demonstrated marked increases in PDGFRa and phospho-AKT expression in cells expressing TW:E12 FDCs and reduced ERK1/2 activation in cells expressing TW:TW FDCs (Figure 4D). The increases in PDGFRa expression and AKT activation are consistent with the TW:E12 generated pro-invasive phenotypes while the reduction in ERK1/2 activity in TW:TW cells is consistent with reduced invasiveness. We, therefore, investigated invasion of cells with overexpression of TW:E12 and TW:TW FDCs. We found that T98G cell invasion was markedly increased (~70%) with expression of the TW:E12 heterodimer while the TW:TW homodimer reduced invasion compared with vector control T98G cells by about 25% (Figure 4E). Using independent primary glioma stem cells G131 we confirmed that overexpression of TW:E12 FDC significant increased cell invasion compared to vector control (Figure S3). Activation of TW:E12 dependent invasion correlated with TW S68 phosphorylation and activation of POSTN expression. We were not able to verify functional effect of TW:TW homodimer in G131, U87 and GBM8 cells due to significant dimer degradation after overexpression. Previously TW:TW homodimer degradation was also noted in lung carcinoma cells [22]. Therefore, we conclude that expression of TW:E12 FDC directly correlates with the level of POSTN expression, the S68 heterodimer phosphorylation and cell invasion. To determine if inhibition of TW phosphorylation is sufficient to inactivate cellular invasion, we repeated invasion assay with cells expressing TW:E12 and mutant TW(S68A):E12 dimers. Our results show that, in the context of FDCs compared to the WT TW:E12 dimer, the TW(S68A):E12 dimer demonstrated reduced potency in the activation of invasion (Figure S4A), which remains higher compared to the vector control. We found that TW(S68A):E12 expressing cells have higher residual levels of POSTN and PDGFRa expression compared to the empty vector control cells (Figure S4B). These results are markedly different from invasive potential of cells expressing TW(S68A) monomer that correlated with downregulation of POSTN expression compared to vector control cells (Figure 1F,G). We speculate that TW(S68A) and E12 fusion proteins produced by the forced dimerization construct are able to generate active dimers.

### 2.4. E12 S139 and TW S68 Phosphorylation Regulate POSTN mRNA Expression

**Figure 5:**
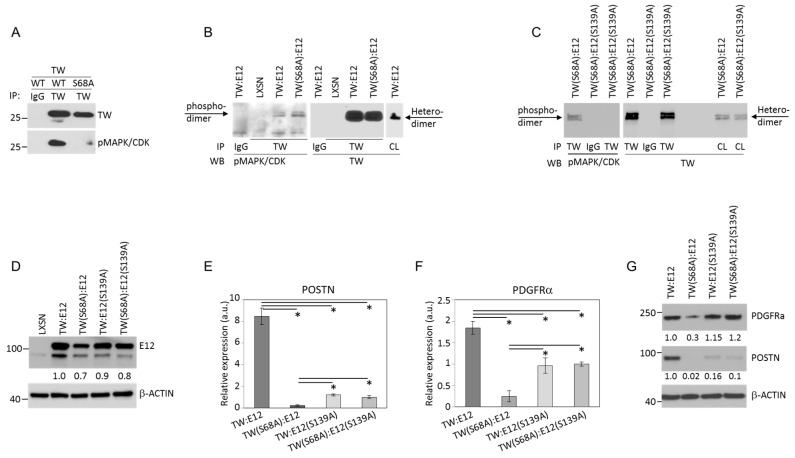
E12 phosphorylation is necessary for regulation of POSTN mRNA expression by TW. (**A**). Confirmation of specificity of pMAPK/CDK substrate antibody. T98G cells with overexpression WT TW or TW(S68A) mutant were immunoprecipitated with TW antibody or nonspecific IgG. Immunoprecipitated proteins were detected using TW or pMAPK/CDK substrate antibody which recognized only WT TW protein but not the mutant form. (**B**) The TW:E12 heterodimer is phosphorylated at S139 harboring a PXS*P consensus phosphorylation site. Nuclear extract from T98G cells expressing TW:E12, TW (S68A):E12, and vector were precipitated using TW antibody. Immunoprecipitated proteins were subjected to Western blot analysis and probed with pMAPK/CDK substrate antibody (left) or TW antibody (right). (**C**) To directly confirm E12 S139 phosphorylation we used cells with TWS68A:E12 or TWS68A:E12S139A dimer overexpression. Proteins immunoprecipitated with TW were probed as in (**B**). IgG: Nonspecific antibody. CL: Whole cellular lysate used as a 2% input control. β-Actin is not shown. (**D**). Comparable levels of expression of wild-type and mutant forms of TW:E12 heterodimers in T98G cells after infection with retroviruses harboring indicated constructs. Whole cell lysates where extracted and probed with E12 or β-Actin antibodies. (**E**,**F**) The mRNA extracted from cell expressing indicated construct was subjected to qRT-PCR using *POSTN* or *PDGFRa* specific primers. The signal was normalized by β-Actin mRNA expression. Target expression in cells with double mutant TW(S68A):E12(S139A) was accepted as 1. *—Differences are statistically significant, *p* < 0.05. (**G**) Confirmation of pTW68-dependent POSTN and PDGFRa proteins expression in cells with overexpression of indicated dimers using Western blot.

We previously reported that TW and POSTN promote glioma cell invasion. Here we showed that TW mediated pro-invasive phenotypes are dependent on the presence of a competent S68 phosphorylation site and E12 hetero-dimerization which in turn upregulates POSTN expression. However, the impact of concurrent phosphorylation of E12 on TW hetero-dimer mediated expression of TW pro-invasive TW target genes like POSTN is unknown. To investigate this we sought for (PXS*P) sites in E12 analogous to that in TW (i.e., S68) reasoning that such sites might be coordinately regulated through a common kinase. Analysis of E12 protein revealed a single candidate consensus phosphorylation site centered at position S139 harboring the same amino-acid sequence as centered at S68 in TW. Since phosphospecific antibody to E12 S139 is not available, we verified the specificity of the pMAPK/CDK substrate antibody to recognize phospho-PXSP sites using TW immunoprecipitated from T98G cells overexpressing WT TW or TW (S68A) mutants. The pMAPK/CDK substrate antibody clearly could not detect mutant TW confirming the specificity of the antibody to recognize only phosphorylated PXS*P (Figure 5A). Next we immonoprecipitated TW:E12 and TW(S68A):E12 fusion proteins. The pMAPK/CDK substrate antibody detected PXS*P site phosphorylation in TW immunoprecipitates from T98G cells expressing both TW:E12 FDC and TW(S68A):E12. Since the latter is not phosphorylated at the (PXS*P) site in TW(S68A), detection of phosphorylation in TW(S68A):E12 FDC expressing cells would indicate phosphorylation attributable only to the S139 (PXS*P) site in E12 (Figure 5B). To directly confirm E12 S139 phosphorylation we immunoprecipitated TW from T98G cells expressing TW(S68A):E12 and TW(S68A):E12(S139A) dimers. Immunoprecipitated dimers were probed with the pMAPK/CDK substrate antibody and phosphorylation was detected in WT E12 protein only (Figure 5C). To determine the functional significance of E12 S139 phosphorylation for target gene expression, we then compared expression of POSTN and PDGFRa in T98G cells expressing TW:E12 FDCs containing hypophosphorylation mutants of TW(S68A) or E12(S139A) alone or together. Comparable levels of WT and mutant FDC expression levels were confirmed by Western blot analysis (Figure 5D). Transcriptional activity evaluated by qRT-PCR quantitation of *POSTN* mRNA revealed that all mutant FDCs dramatically inhibited *POSTN* mRNA expression (Figure 5E). Similarly, we observed inhibition of *PDGFRa* mRNA expression in cells expressing TW(S68A):E12 mutant dimers compared to cells expressing WT TW:E12 dimers (Figure 5F). While E12(S139A) single and double mutants abrogate TW S68 phosphorylation-dependent gene regulation, we observed increased target gene expression compared to the TW(S68A):E12 single mutant. Observed *POSTN* mRNA expression regulation correlated with levels of intracellular protein expression measured by Western blot (Figure 5G). Clear pTWS68-dependent PDGFRa protein expression was detected. However, no protein expression reduction was detected in cells with overexpression of TW:E12(S139A) or TW(S68A):E12(S139A) dimers, which is consistent with less than two-fold downregulation of *PDGFRa* mRNA expression. We speculate that target genes might be regulated by alternative transcription factors. Further investigations are needed to determine precise role of pE12S139-dependent regulation. Together these results provided the first evidence that regulation of a TW target gene is dependent not only on its intrinsic phosphorylation status but also that of a heterodimer partner, E12.

## 3. Discussion

TW and its transcriptional target POSTN [26] comprise an important signaling axis that promotes mesenchymal phenotypes in cancers including GBM [7,8,10,11,24,27,28]. Little is known regarding mechanisms by which TW regulates POSTN yet their elucidation could be critical for devising therapeutic strategies to disrupt their functional effects in cancer. Prior observations established the importance of site-specific TW phosphorylation [13,14,15,29] and TW dimerization motifs for malignancy [22,23,30,31], but their potential interactions, particularly in the context of POSTN regulation and mesenchymal phenotypes, have not been well characterized. Here we focused on the role of TW serine 68 phosphorylation to address the hypothesis that TW site-specific phosphorylation influences TW dimerization and downstream expression of pro-invasive targets, such as POSTN in GBM cells. Our results demonstrated a mechanistic linkage between TW phosphorylation and preferential formation of specific dimerization motifs that regulate pro-invasive gene expression. Furthermore, we provide evidence that a novel mechanism involving coordinated phosphorylation of TW and E12 also regulates POSTN expression. This new understanding has potential significance for development of therapeutic strategies to disrupt TW signaling in GBM and other cancers where TW regulates tumor malignancy.

Several lines of evidence support the importance of a TW-POSTN signaling axis in cancer. Consistent with the observation in normal mouse cells that TW is a direct transcriptional regulator of *POSTN* [26] changes in POSTN expression are directly related to TW in gain and loss of function in glioma cells [24]. Of clinical and functional relevance, TW and POSTN expression are highly correlated and predictive of survival in GBM patient samples and both promote highly overlapping pro-invasive and tumorigenic phenotypes in glioma cells [11,24]. However, the mechanisms by which TW regulates POSTN expression are largely unknown. Given that TW functions relevant to EMT are regulated through site-specific phosphorylation (such as S68) and TW dimer motifs in other cancers we sought to define their potential roles in regulating POSTN expression and invasive phenotypes in GBM.

Using mass spectrometry and Western blotting TW S68 phosphorylation was confirmed in GBM cells and importantly in patient derived GBM samples. Over-expression of TW(S68A) hypo-phosphomutant markedly reduced TW mediated invasion of T98G cells indicating a role for TW S68 phosphorylation in GBM cell invasion. Similarly, Hong et al. (2011) reported inhibition of invasion of MCF-7 breast carcinoma cells expressing TW(S68A) vs. WT TW [14]. Consistent with this dephosphorylation of TW S68 by small C-terminal domain phosphatase 1 (SCP1) in breast cancer cells also markedly attenuates TW-mediated invasion [15]. Together these observations support the importance of serine 68 phosphorylation as a determinant of TW mediated invasion in multiple cancers. In addition to pro-invasive function, we also showed that pTWS68 enhanced formation of TW:E12 heterodimers which, in contrast to TW homodimers, promoted POSTN expression, invasion, and alterations in cellular architecture and adhesion. Using complementary co-IP and affinity purification analyses we demonstrated that phosphorylation of TW S68 promoted TW dimerization with E12 and that expression of TW(S68A) in T98G cells dramatically reduced TW interactions with E12 without apparent impact on the formation of TW(S68A) homodimers. Of note, phosphorylation of TW at S123, T148, S184 enhances malignant phenotypes in pancreatic carcinoma cells in concert with regulation of TW homodimer motifs [23]. Together with our current findings, this study supports the potential importance of site-specific TW phosphorylation in regulating interaction with specific dimerization partners. By contrast, Hong et al. (2011) were unable to document formation of TW:E12 heterodimers in a mammalian two-hybrid assay when expressing wild-type or TW(S68A) in Hela cells. [14]. This disparate result may reflect different analytic methodologies and cell type specific mechanisms that require additional investigation in a wider range of cancer cells to reconcile.

The significance of mechanisms that regulate TW:TW vs. TW:E12 dimer formation is underscored by the robust functional impact of specific TW dimer motifs which serve as phenotypic switches in cancer and development. With the one exception as noted above [23], the TW:E12 heterodimer, as opposed to TW homodimer, has been implicated in increased malignancy in several cancers in part through activation of EMT like phenotypes [22,30,31]. In mouse Myc-CaP prostate carcinoma cells TW:E12 FDCs, but not TW homodimer FDCs, promote EMT phenotypes [30], while in hTERT immortalized human mammary epithelial cells (MECs) TW:E12 FDCs overcome RAS-induced senescence and promoted tumorigenesis compared with TW:TW FDCs [22]. In NSCLC cell lines TW:E12 FDCs activated the expression of EMT-related genes SNAI2 and YBX1 and rescue harmine-induced cytotoxicity in vitro [31]. In BxPC3 pancreatic carcinoma cells Aurora kinase-A mediated phosphorylation of TW at S123, T148 and S184 promote increased malignant phenotypes and inhibit TW heterodimerization with E12 and Hand2. While this may indicate a preference of TW homodimerization, TW-TW homodimerization was not directly validated so the possibility that TW was interacting with alternative known heterodimer partners (e.g., TCF4 or TCF12) cannot be excluded [23]. Together with our results showing dramatic differential effects of TW:E12 vs. TW:TW dimers on activation of pro-invasive gene expression (*POSTN*/*PDGFRa*), signaling pathways (ERK/AKT) and mesenchymal phenotypes, these studies demonstrate the importance of TW dimerization as a phenotypic switch regulating cancer malignancy.

In cancer studies TW dimerization has not been linked to regulation of POSTN expression, however, in mouse cranial suture development, TW homodimers upregulate POSTN in the osteogenic front [17]. This is in contrast to our findings, where TW:E12 heterodimers upregulated, and TW homodimers downregulated, *POSTN*. These differences suggest that additional factors, perhaps at the promoter level, may be involved in TW dimer specific regulation of *POSTN*. The *POSTN* promoter contains TW e-box sites, yet detection of TW interactions at the *POSTN* promoter has not been consistent [26,32]. Together these observations indicate that additional study is needed to define the specific requirements for TW dimer activation of *POSTN* and other mesenchymal target gene expression at the transcriptional level.

Here we also presented the novel finding that TW:E12 dimer regulation of *POSTN* and *PDGFRa* expression was dependent on the capacity for phosphorylation of a PXSP site in E12 (S139) analogous to that in TW S68. While loss of phosphorylation capacity for the S139 PXSP site either in E12 alone or in TW:E12 double mutant dimer (TW(S68A)):E12(S139A)) was clearly sufficient to significantly inhibit *POSTN* expression, expression was not maximally reduced to the levels achieved by expression of TW:E12 FDCs with TW(S68A) mutant alone. Further studies to discern their unique effects on recruitment of transcriptional co-factors or engagement of the *POSTN* promoter may provide insight into the mechanisms underlying these observations. Regardless, this finding provides the first indication of a potential broader scope of TW regulation, not only through its site-specific phosphorylation but also through that of its binding partners. Given that TW dimer motifs function as potent transcriptional and phenotypic switches it is of interest to determine whether TW or other bHLH dimerization preferences are encoded through coordinated phosphorylation at analogous sites. If so, targeting the common upstream kinases may prove to be useful therapeutic strategies to inhibit TW dimer specific malignant phenotypes. For example, inhibition of MAP kinases that phosphorylate PXSP sites (JNK, ERK, and p38), reduced expression of pTWS68, and total TW [14].

In summary, the current study demonstrated a novel mechanism by which site-specific TW S68 phosphorylation and dimerization motifs promoted glioma cell invasion with concomitant upregulation of pro-invasive genes. TW and E12, but not TW, homodimerization activated pro-invasive phenotypes and molecular changes in glioma cells. The TW and E12 heterodimer activated POSTN, PDGFRa, FAK expression, AKT phosphorylation, and cytoskeletal changes consistent with pro-invasive cellular phenotype. We further showed that in the context of FDCs, TW:E12 heterodimers are fully transcriptionally active only in the presence of phosphorylation-competent sites at S139 in E12 and S68 in TW. For the first time we show that E12 S139 is required for TW S68-dependent regulation of *POSTN* and *PDGFRa* mRNA expression suggesting broad transcriptional relevance of the mechanism. Therefore, targeting TW:E12 heterodimerization through dimer disruption or dephosphorylation could be therapeutically relevant for GBM treatment. Future studies will validate the scope of these mechanisms in additional glioma cell lines by analysis of direct *POSTN* promoter interactions, effects of kinase inhibition and establishing in vivo phenotypes in xenograft models.

## 4. Materials and Methods

### 4.1. Cells and Tissue

Established human GBM cell lines (T98G and U87) were maintained in Dulbecco’s modified Eagle’s medium/F12 with 10% fetal bovine essence (VWR, Radnor, PA, USA), while human GBM8, GBM4, and G131 stem-like cell line (GSC) [33] were maintained in serum-free Neurobasal medium supplemented with N2, B27 (Invitrogen, Carlsbad, CA, USA), FGF, and EGF (Peprotech, Rocky Hill, NJ, USA). T98G and U87MG cells were transduced with retrovirus, while GSCs were transduced with lentivirus to express indicated transgene or empty vector. U87 (dTW-A) with CRISPR mediated deletion of TW and U87 (dTW-A) + TW cells with subsequent overexpression of exogenous TW were previously described [24]. These cells demonstrated increased malignancy in vivo and were isolated from tumors to model primary patients primary human glioma cells. Human glioma tumor samples were acquired according to a protocol (00002162) approved by the Institutional Review Board of the Human Subjects.

### 4.2. Expression Constructs and Virus Production

TW expression constructs were described previously [10]. QuickChange site directed mutagenesis kit (Agilent, Santa Clara, CA, USA) was used to replace Serine at position 68 in TW and/or position S139 in E12 to alanine. E12 monomer was amplified from plasmid provided by Dr. Tapscott [34] and cloned in TA cloning vector (Invitrogen, Carlsbad, CA, USA). Resulting product was transferred into pLXSH retroviral vector [35]. To obtain NTAP expression construct TW or TW(S68A) were fused in frame with NTAP tag provided by Dr. Gingras [36], followed by transferring the entire tagged sequence into the pLXSN retroviral vector [10]. Tethered TW:E12 and TW:TW dimer constructs were built by linking in frame TW without stop codon with E12 or TW using a previously described linker peptide [37], followed by cloning into pLXSN or lentiviral pWPI-neo expression vectors (Addgene, Watertown, MA, USA). The TW(S68A)-HA construct was generated by PCR and cloned into pLXSH. All constructs were confirmed by sequencing. To knockdown TW and E12 commercially available Mission shRNA lentiviral vectors were used (MilliporeSigma, St. Louis, MO, USA). Amphotropic retroviruses were produced by transfection in Phoenix cells. Lentiviruses were produced by transfection expression construct and packaging DNA in 293FT cells (Thermo Fisher Scientific, Waltham, MA, USA). Next day transfection media was changed and viruses were harvested 24, 48 and 72 hours and filtered through 0.4 µM PVDF syringe driven filter. Target cells were infected in the presence of 10 ug/mL of polybrene (MilliporeSigma, St. Louis, MO, USA). To infect GBM8 cells lentiviruses were harvested in serum-free conditions. Transduced cells were selected with appropriate antibiotics.

### 4.3. Antibody Validation

Antibodies used for Western blot (TW, E12 (Santa Cruz, Dallas, Texas, USA), TW rabbit antibody and POSTN rabbit antibody were provided by Dr. Glackin and Dr. M. Zhu, respectively) were validated on the Western blot by detection of target protein overexpression or knockdown. HA-tag antibody was validated by overexpression of HA-tagged TW protein. Phospho TW S68 antibody (Abcam, Cambridge, UK) was validated by overexpression of WT TW vs. TW with mutation of Serine at position 68 to alanine. Phospho-MAPK/CDK antibody (Cell Signaling, Danvers, MA, USA) was validated by immunoprecipitation of WT and S68A mutant TW protein followed by detection of phosphorylation by Western blot.

### 4.4. Immuno-Precipitation and Affinity Purification

Nuclear extracts were isolated as previously described [38] and diluted to achieve final concentration 150 mM of NaCl. Total proteins were isolated using lysis buffer: 20 mM Tris-HCl (pH 7.6), 150 mM NaCl, 1 mM Na_2_EDTA, 1 mM EGTA, 1% Triton, 2.5 mM sodium pyrophosphate, 1 mM beta-glycerophosphate, 1 mM Na_3_VO_4_, 1 mM sodium fluoride, and 1 mM PMSF. After pre-clearing protein preparations were incubated with primary antibody overnight followed by incubation with Protein A or G agarose (Santa Cruz, Dallas, TX, USA) as appropriate. For affinity purification nuclear extracts from cells expressing NTAP tag fusion proteins were incubated overnight with agarose beads conjugated with rabbit immunoglobulin (MilliporeSigma, St. Louis, MO, USA). Immuno- and affinity precipitates were washed four times with washing buffer, resuspended in the RIPA buffer, vortexed, and heated to elute proteins. Gel loading buffer with β-mercaptoethanol was added to resolve proteins on the Western blot.

### 4.5. Invasion, Adhesion Assays, and Immunofluorescence

Three independent invasion assays with three technical replicates were performed as previously described [10,11]. Invasion inserts after processing were stained to visualize invading cells. Stained inserts were scanned using a DMi8 microscope (Leica, Buffalo Grove, IL, USA). Cells were counted using the image analysis module in LAX software (Leica). Results are presented as a mean percent change over control for each independent assay. For adhesion assay 96-well plates were coated with recombinant fibronectin (5 µ/mL). Wells were blocked with freshly-prepared 0.5% BSA in serum free media. T98G cells with dimer overexpression (2 × 10^4^ cells/mL) were allowed to adhere for 1 h. Cells were washed to remove floating cells, fixed and stained with 1% crystal violet (MilliporeSigma, St. Louis, MO, USA). After cell lysis, absorbance was quantified at 550 nm. Differences in cell adhesion are shown as the fold change over T98G control cells accepted as 1. Adhesion assays were performed with five technical replicates. Three separate experiments were analyzed and the significance of differences was determined by Student’s t-test. Results are presented as adhesion fold change over mean control accepted as 1. F-actin filaments were stained with phalloidin conjugated with Texas Red. Focal adhesion kinase was detected using FAK antibody (Upstate Biotechnology, NY, USA) followed by secondary antibody Alexa Fluor 488 (Invitrogen, Carlsbad, CA, USA). Slides counterstained with DAPI were imaged using a DMi8 microscope to collect single cell images for quantitative analysis of FAK. To analyze the FAK distribution we used a MATLAB script to detect the dots in cells using segmentation method [11].

### 4.6. Quantitative Real Time PCR 

RNAeasy kit (Qaigen, Germantown, MD, USA) with on column DNAse digest was used for total RNA extraction. A reverse transcription kit (Clontech, Mountain View, CA, USA) was used to reverse transcribe 1 µg of total RNA. Quantitative PCR amplifications were performed using SYBR Green master mix in CFX380 (Bio-Rad Laboratories, Hercules, CA, USA) and preset standard amplification cycle for 40 rounds. Following primers were used for *POSTN* amplification: forward-AATCATCCATGGGAACCAGA, reverse-ATTGGTGGGAGCAAAGAGTG. Premade primers for *PGDFRa* amplification were purchased (MilliporeSigma).

### 4.7. Mass Spectrometry

The precipitated proteins were trypsinized directly off beads following reduction with 5 mM Dithiothreitol and alkylation with 15 mM iodoacetamide. Following “stage-tip” desalting [39], peptides were resuspended in binding buffer (80% acetonitrile, 0.2% trifluoroacetic acid) and incubated for 30 min with immobilized metal affinity chromatrography (IMAC) beads. Phosphorylated peptides were washed three times with binding buffer, eluted with 1.4% ammonium hydroxide, and processed for mass spectrometry. Samples were analyzed on a Velos-Pro/Orbitrap-Elite (Thermo Fisher Scientific, Waltham, MA, USA) hybrid mass spectrometer. Raw mass spectrometry data was searched with SEQUEST and phosphorylation was queried via specification of a differential modification of 79.6 atomic mass units on serine, threonine, or tyrosine. Proteins were scored using the Institute for Systems Biology trans-proteomic pipeline [40], and the TWIST1 phosphopeptide was manually verified. The immune-purified TWIST1 from GBM8 and U87 (dTW-A) with TW overexpression cell lines were analyzed in a separate facility to validate the previous result. The purified proteins from three independent preparations were subject to SDS-PAGE gel and in-gel digestion using trypsin and analyzed by LTQ Orbitrap Elite. Data analysis is done using a Mascot algorithm (Mascot 2.4, Matrix Science, Boston, MA) with the same parameters as above.

### 4.8. Statistical Analysis

An unpaired two-sided t-test was used to calculate the *p* value. Results are presented as the mean ± s.e.m. Images were prepared using ImageJ, Adobe Creative Suite (San Jose, CA, USA), and Microsoft Excel (Microsoft, Redmond, WA, USA).

## 5. Conclusions

The current study demonstrated a novel mechanism by which site-specific TW S68 phosphorylation and dimerization motifs promoted glioma cell invasion with concomitant upregulation of pro-invasive genes. Pro-invasive TW and E12 heterodimerization is regulated by TW S68 phosphorylation. We further showed that in the context of forced dimerization constructs, TW:E12 heterodimers are fully transcriptionally active only in the presence of phosphorylation-competent sites at S139 in E12 and S68 in TW.

## Supplementary Materials

The following are available online at https://www.mdpi.com/2072-6694/11/9/1392/s1, Figure S1. Identification of TW S68 phosphorylation by mass spectrometry; Figure S2. Quantification of relative fraction of phosphorylated TW in U87dTW-A and GBM8 cells with TW overexpression; Figure S3. TE:E12 dimer over-expression in G131 primary glioma cells promotes invasive phenotype; Figure S4. Invasion of T98G cells with overexpression of empty vector, WT TW:E12 and TW(S68A):E12 dimers.
