# Supplementary material for: TWIST1 Heterodimerization with E12 Requires Coordinated Protein Phosphorylation to Regulate Periostin Expression
Source: Cancers (Basel). 2019 Sep 18;11(9):1392. doi: 10.3390/cancers11091392 (PMC6770789; doi:10.3390/cancers11091392)
Supplement: Supplementary file 1 [file cancers-11-01392-s001.zip › cancers-570996 supplementary final/cancers-570996-supplementary final.pdf]

cancers

Supplementary Materials

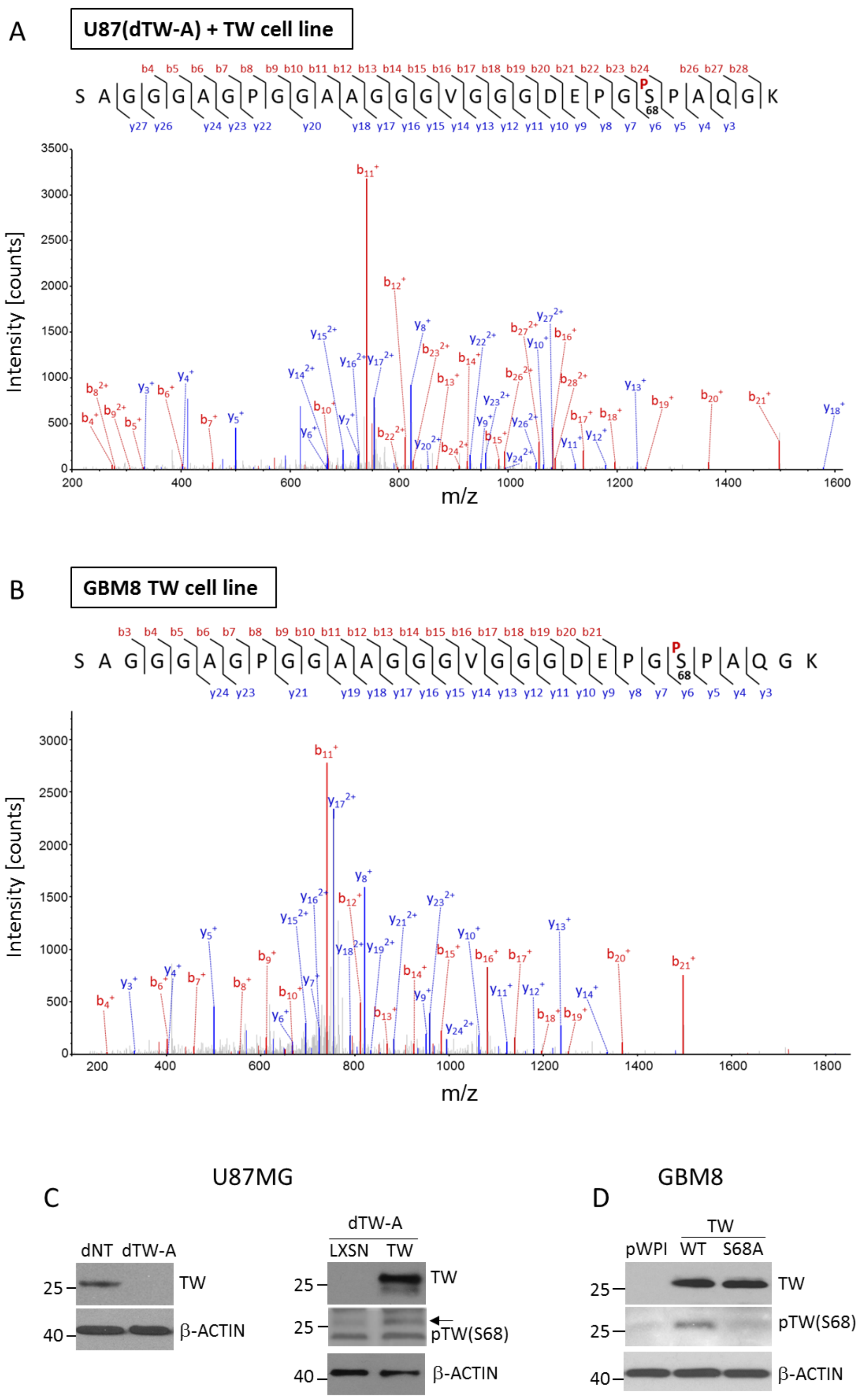

Figure S1. Identification of TW S68 phosphorylation by mass spectrometry. Nuclear lysates from U87(dTW-A) + TW (**A**) and GBM8 over-expressing TW (**B**) were immuno-precipitated using TW

antibody. The spectra of the TWIST1 S68 containing peptide and phosphorylated serine at position 68 are shown. b- and y-ion fragments are indicated on the spectrum with their observed masses (m/z). For U87(dTW-A) + TW cells (**A**) ions fragments b24, b26-b28 and y6-y18, y20, y22-y24, y26, and y27 correspond to phosphorylated Serine. In GBM8 cells (**B**) y-ion fragments (y6-y19, y21, y23, y24) identified S68 phosphorylation. (**C**) Confirmation of TW deletion (left), subsequent TW overexpression and S68 phosphorylation (right) in total extracts from U87MG cells. (**D**) Total protein lysates from GBM8 cells with TW, TW(S68A) or empty pWPI vector overexpression were probed with a pTW S68 specific antibody. β-Actin antibody was used as an endogenous control.

A

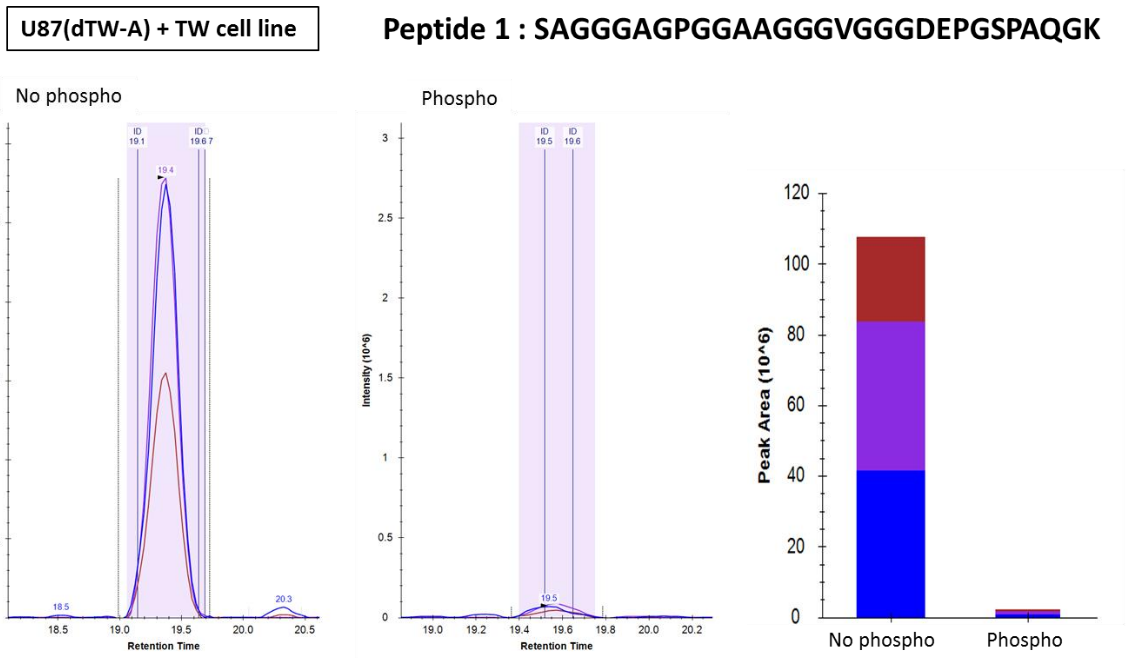

B

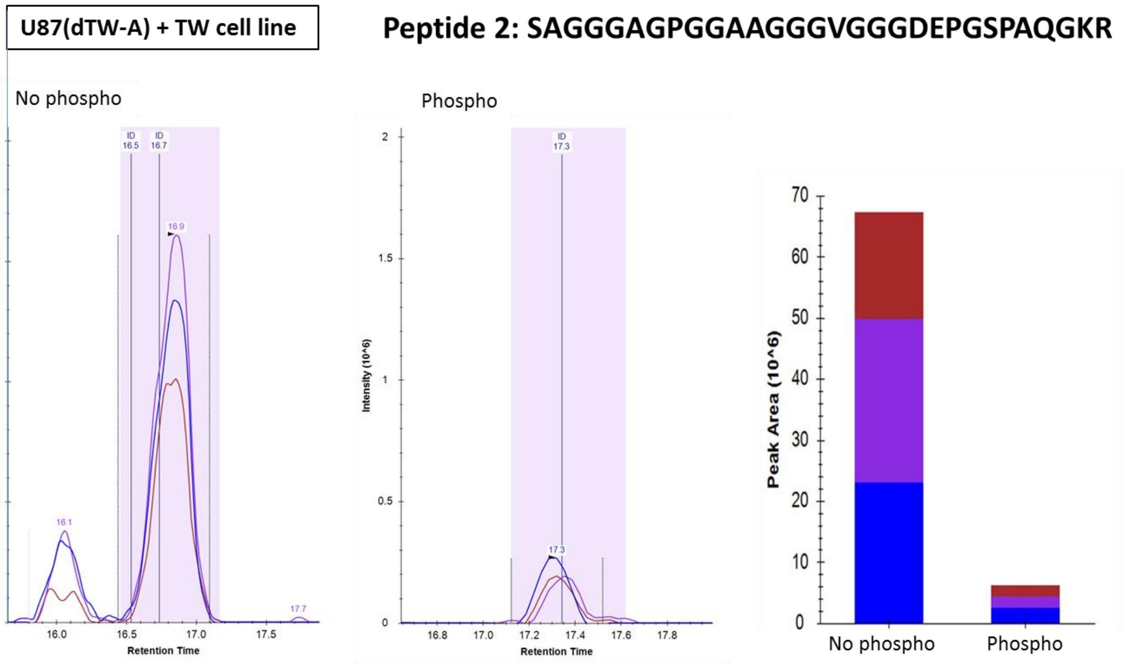

Figure S2. *Cont.*

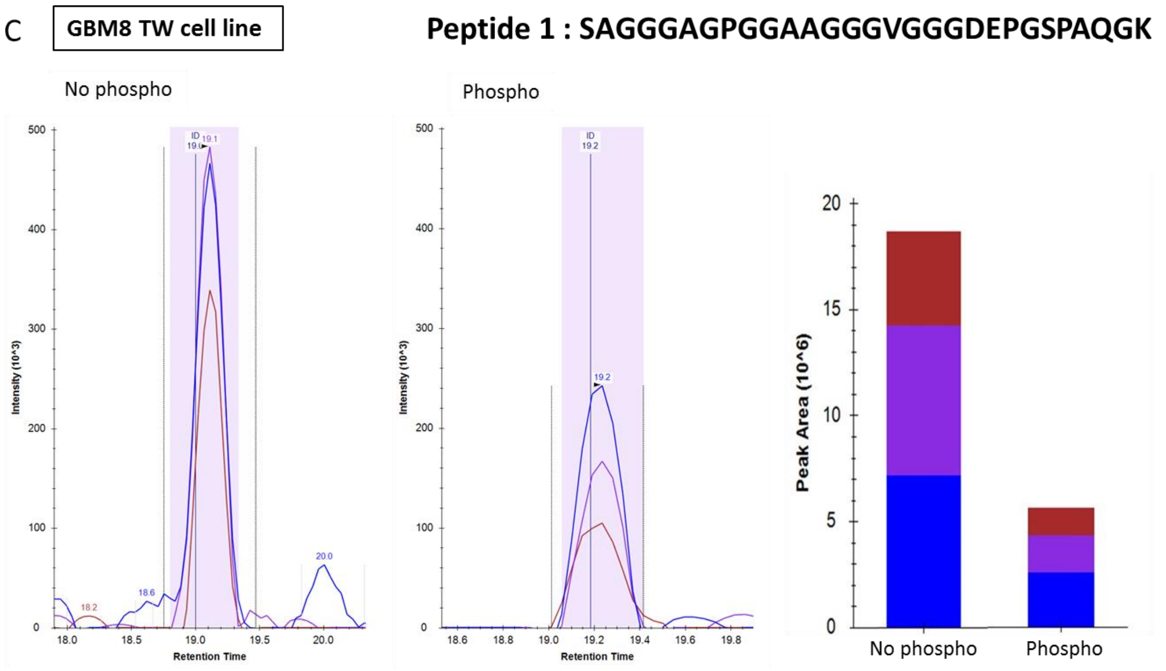

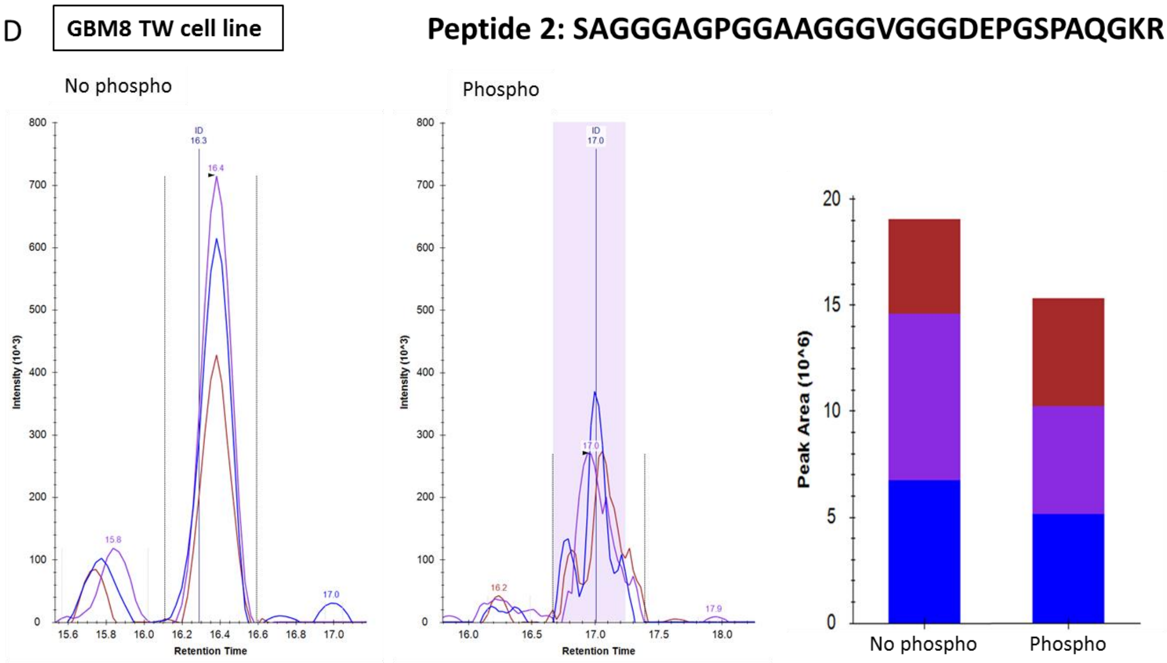

Figure S2. Quantification of relative fraction of phosphorylated TW in U87dTW-A and GBM8 cells with TW overexpression. Area-under-curve of full mass (MS1) from phosphorylated (phospho) and non-phosphorylated (no phospho) peptides are shown in chromatogram. Bar diagram shows peak areas of three measurements for one biological replicate. One representative result from total of three independent replicates is shown.

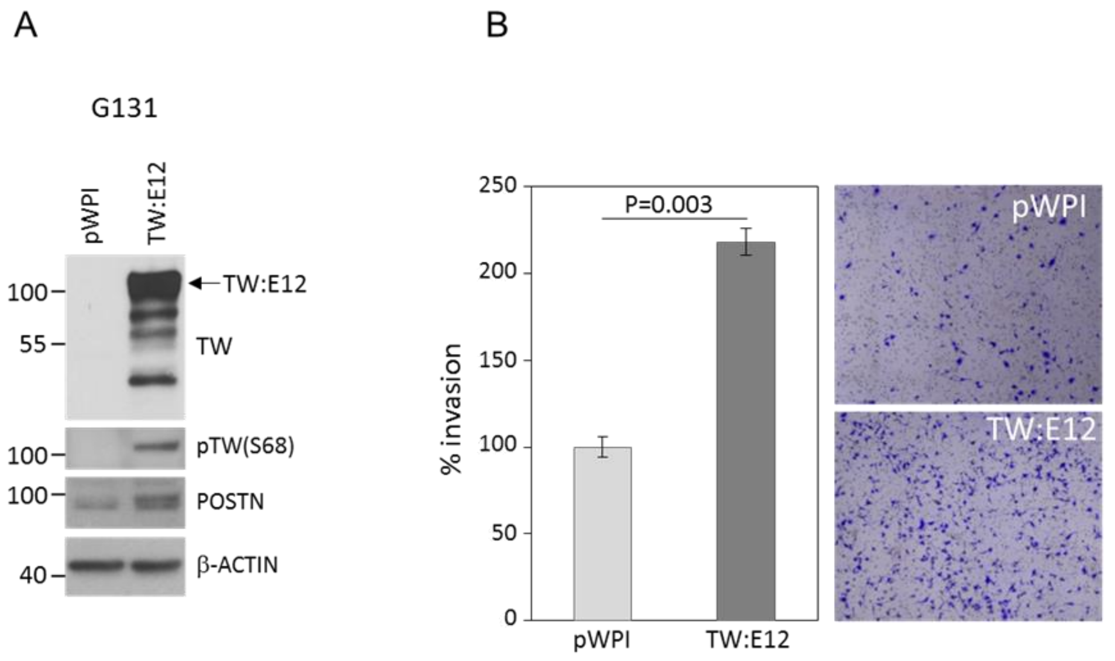

Figure 3. TE:E12 dimer over-expression in G131 primary glioma cells promotes invasive phenotype. (**A**) Confirmation of TW:E12 FDC overexpression, dimer phosphorylation and POSTN activation in G131 cells. Dimer position is shown with arrow. (**B**) Activation of invasive phenotype of G131 cells with TW:E12 heterodimer overexpression in vitro.

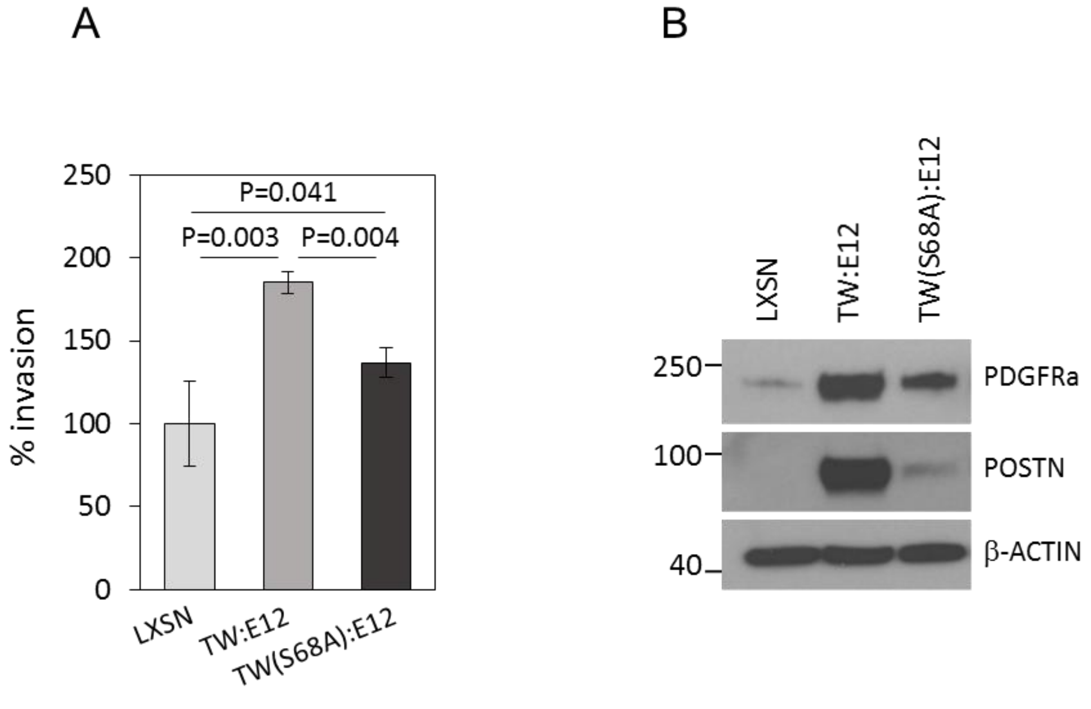

Figure S4. Invasion of T98G cells with overexpression of empty vector, WT TW:E12 and TW(S68A):E12 dimers. **Table 68.** expressing cells were significantly less invasive (~48% reduction) compared with TW:E12 but ~37% greater than empty vector control cells. The reduction in invasion correlated with a marked decrease in POSTN and PDGFRa expression in cells expressing TW(S68A):E12 vs WT TW:E12 cells. However levels of target expression in TW(S68A):E12 cells remained greater than that observed in empty vector control cells (LXSN). Blots are overexposed to demonstrate differences in target protein expression among all three cell lines.
