# Supplementary figures and images for: TWIST1 Heterodimerization with E12 Requires Coordinated Protein Phosphorylation to Regulate Periostin Expression
Source: Cancers (Basel). 2019 Sep 18;11(9):1392. doi: 10.3390/cancers11091392 (PMC6770789; doi:10.3390/cancers11091392)

Fig.1

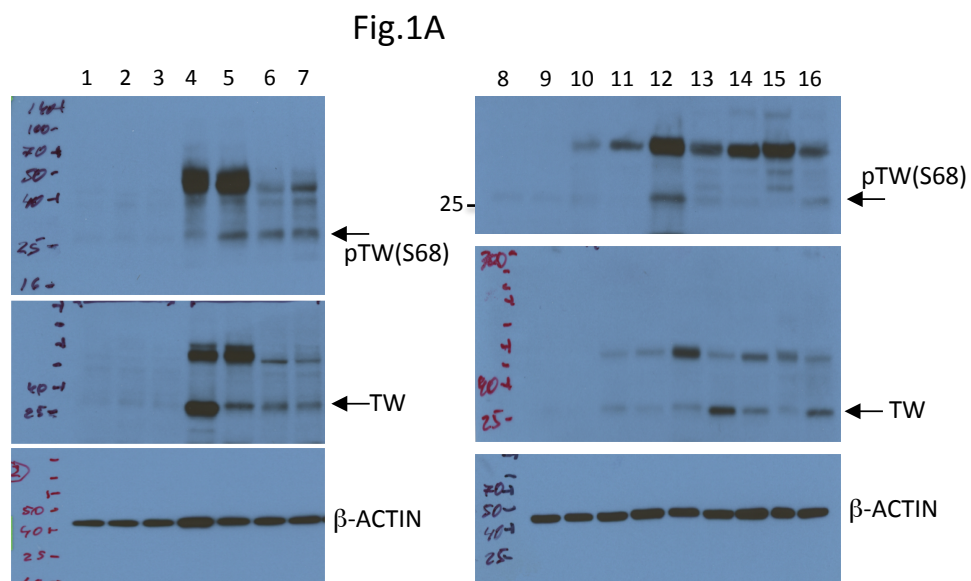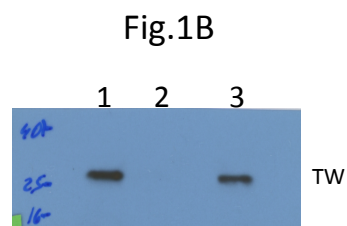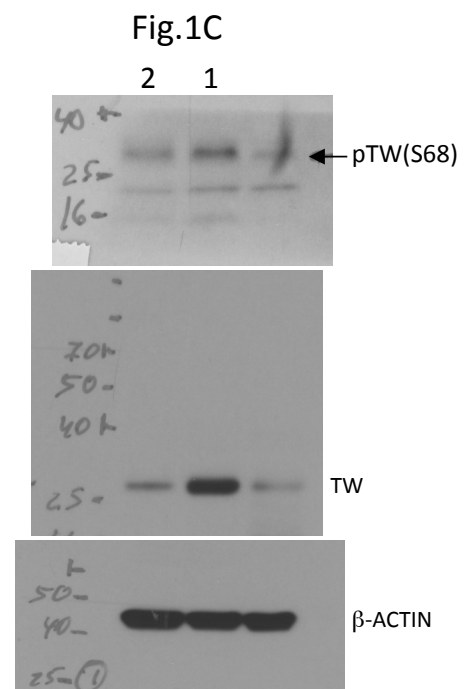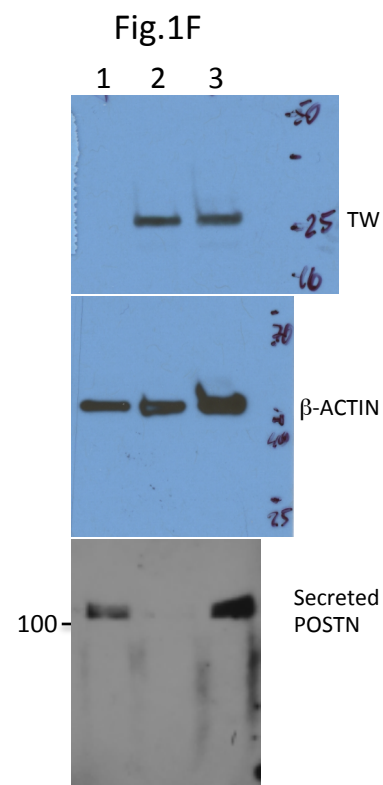

Fig.2

Fig.2A

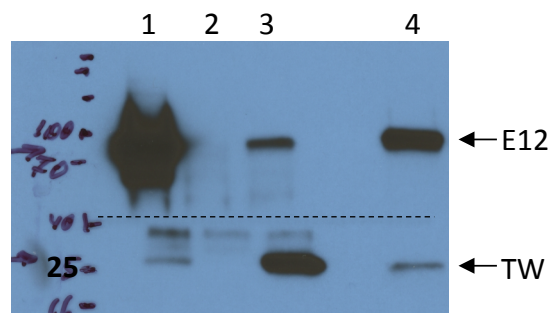

Fig.2B

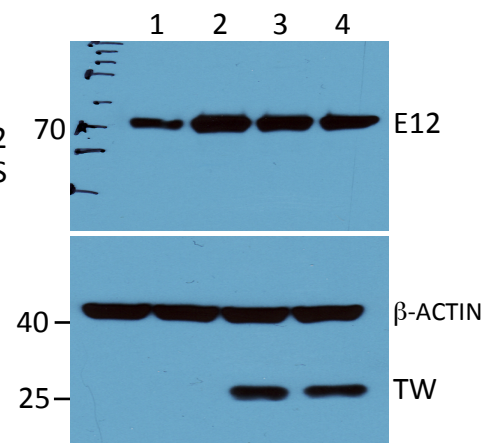

Fig.2C

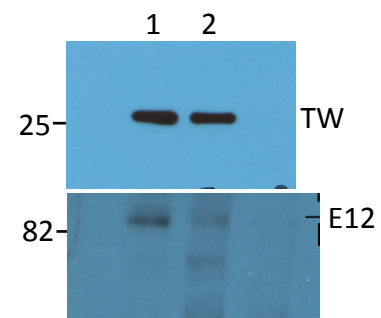

Fig.2D

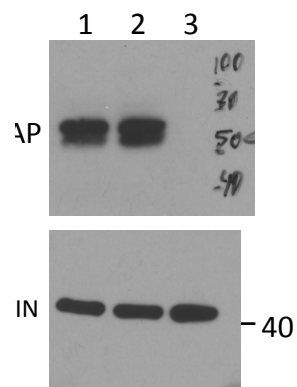

Fig.2E

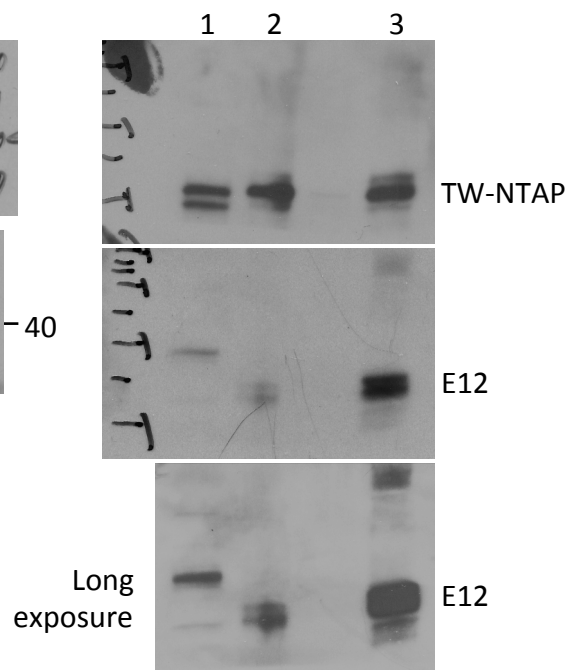

Fig.2F

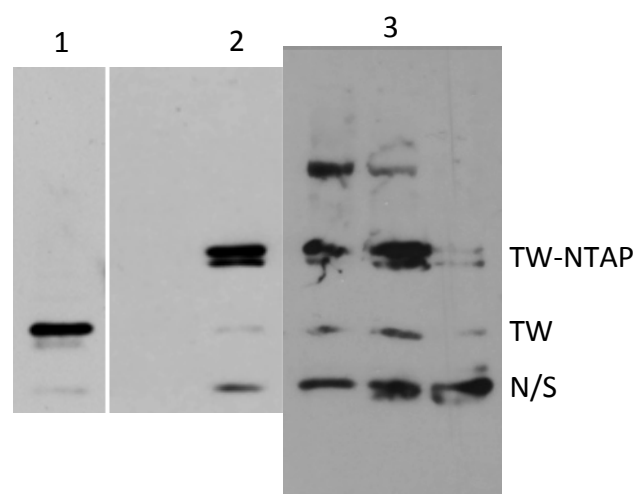

Fig.2G

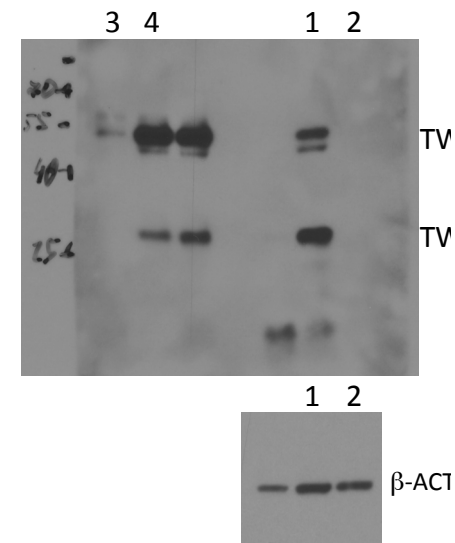

Fig.3

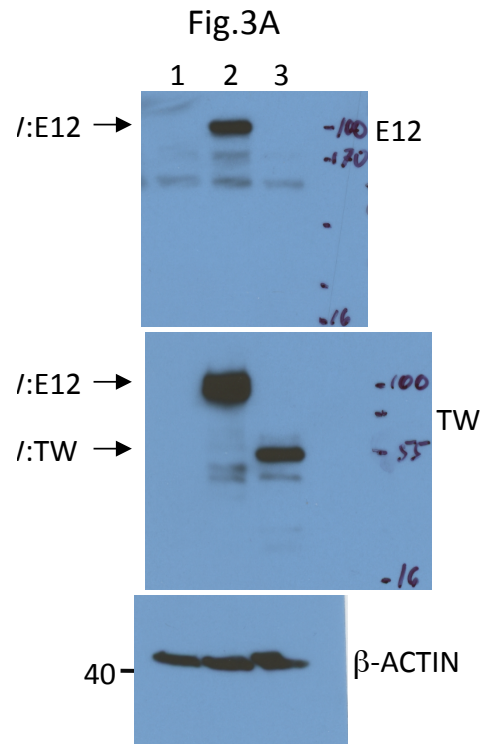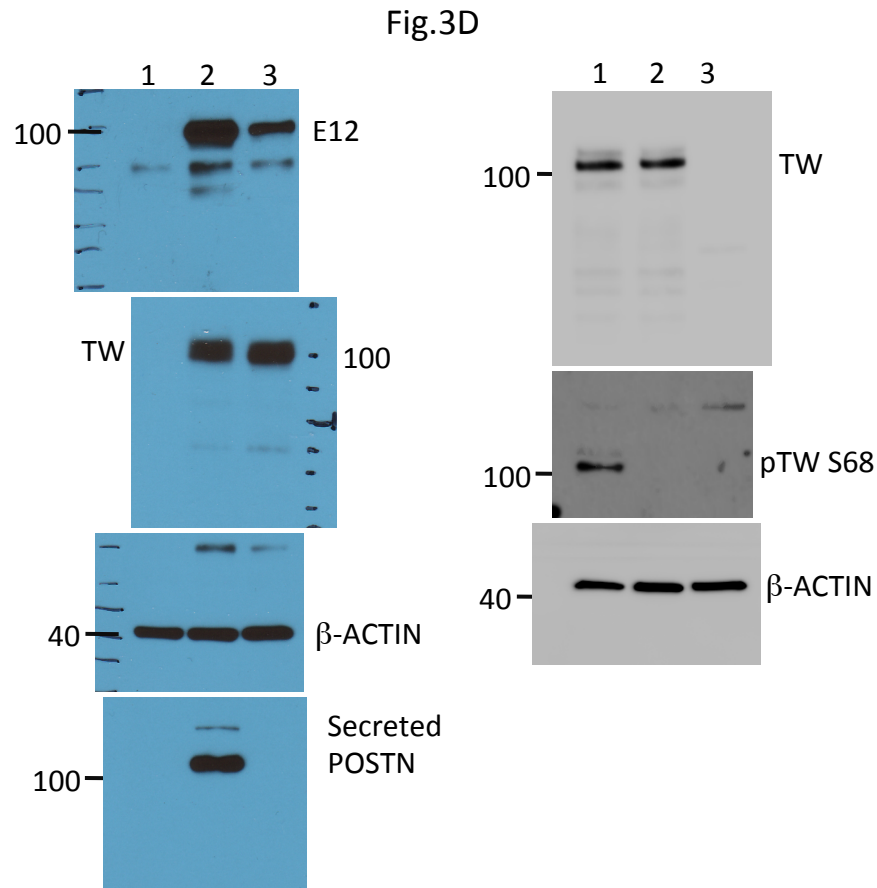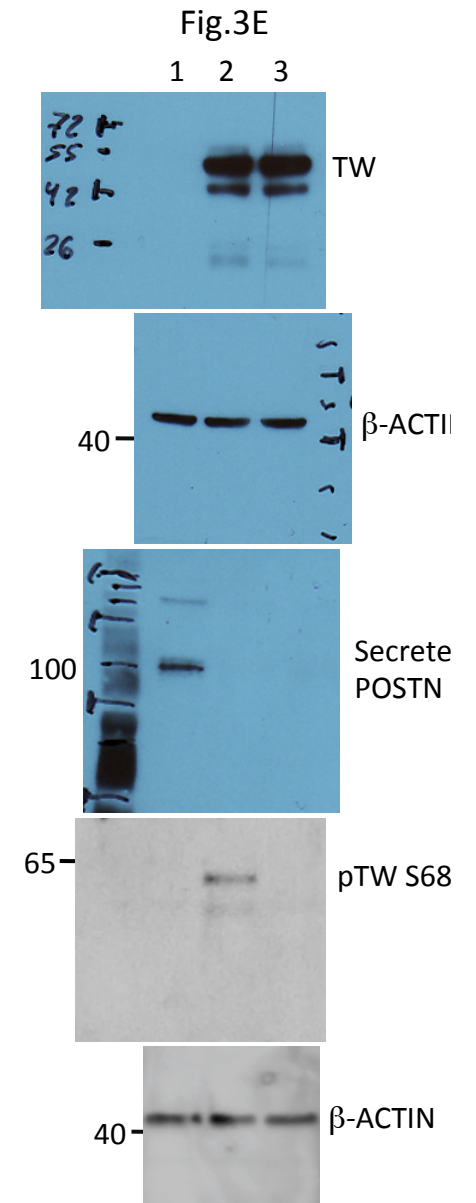

Fig.3

Fig.3F

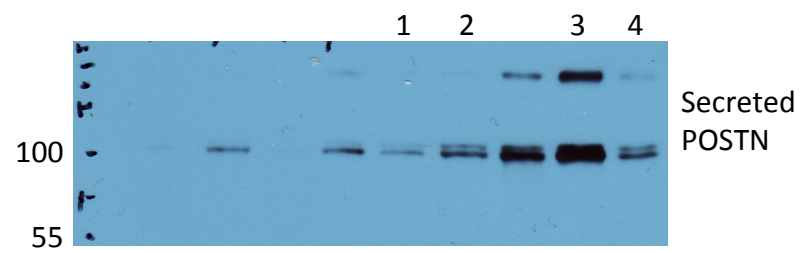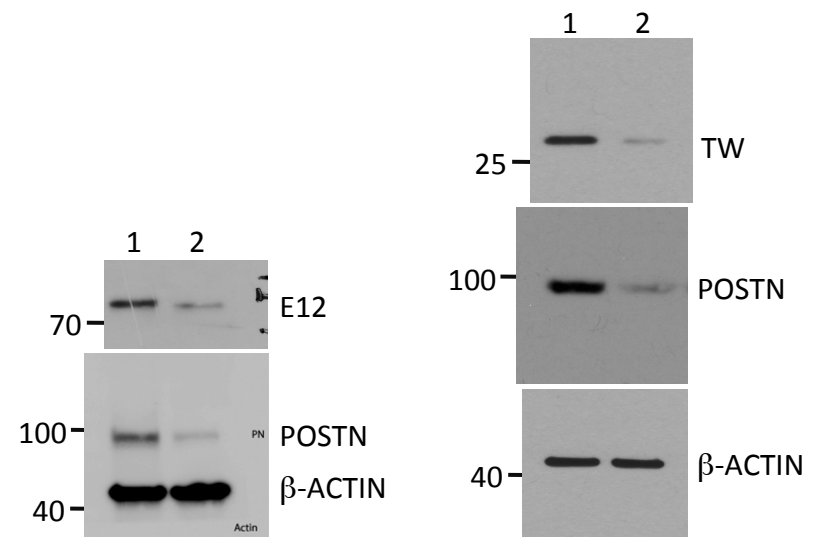

Fig.4

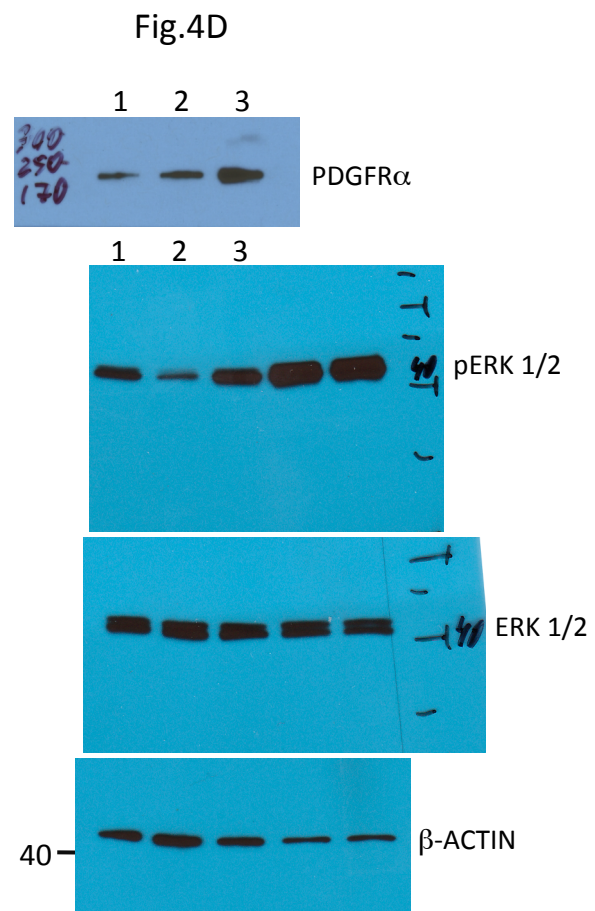

Fig.5A

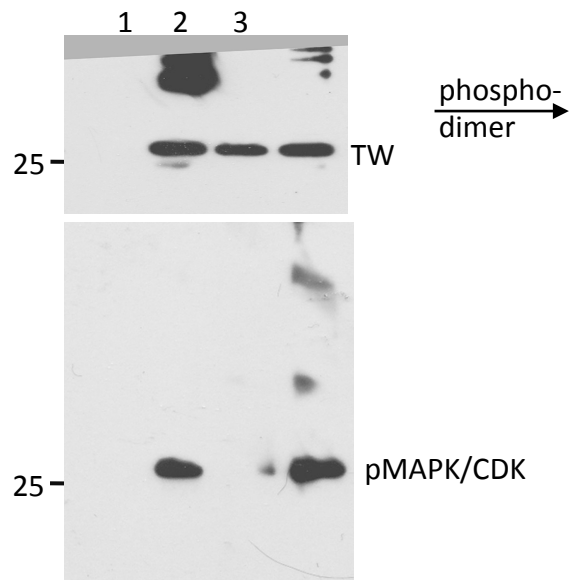

Fig.5B

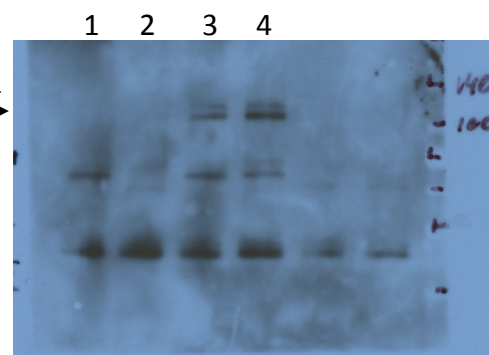

Fig.5

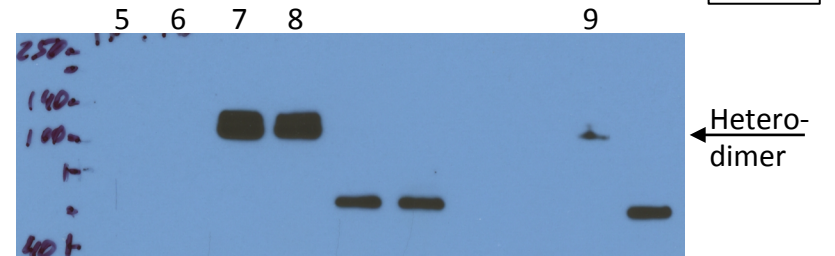

Fig.5C

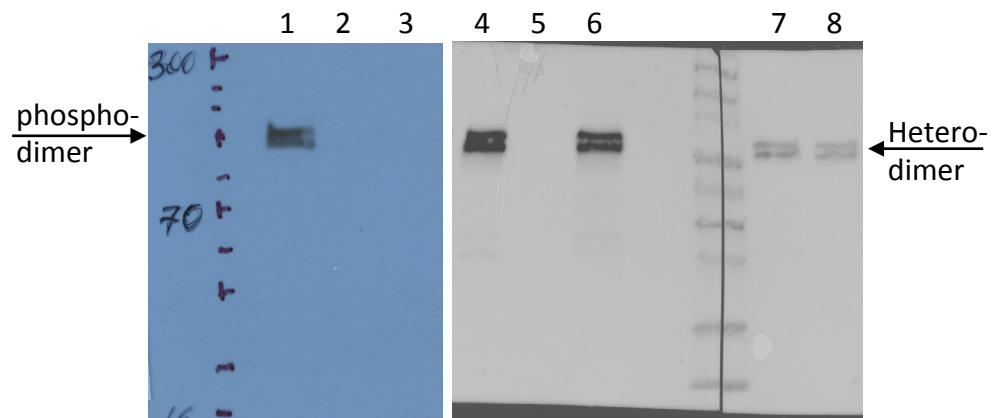

Fig.5D

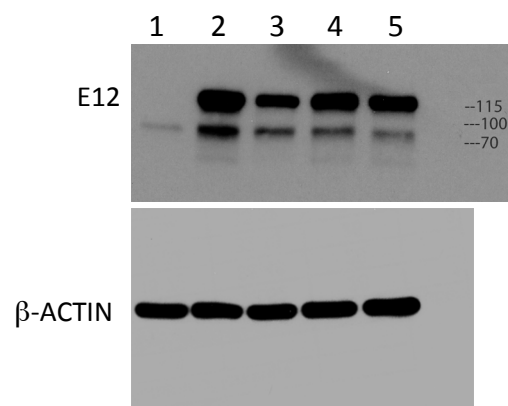

Fig.5G

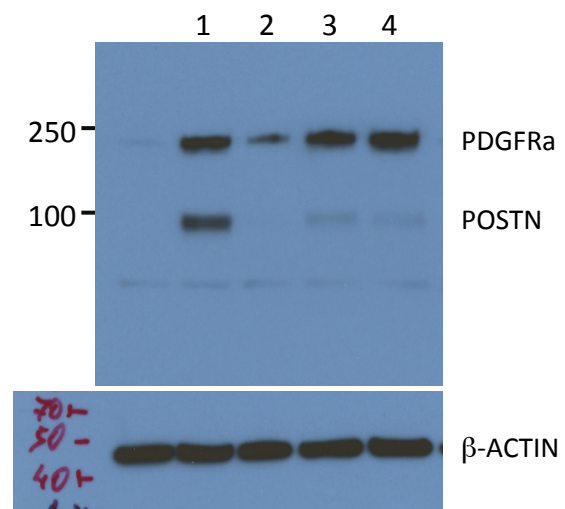

Supplement: Supplementary file 1 [file cancers-11-01392-s001.zip › cancers-570996 supplementary final/cancers-570996-western blot figures.pdf]
